# Dietetic Gazette

**Published:** 1891-02

**Authors:** 


The Dietetic Gazette. A monthly journal of physiological medicine. New York: P. O. Box 2898. Price, $1.00 a year.

The December number of this instructive periodical is before us, and is well filled with valuable articles upon dietetics. Thus we observe an article by Allan McLane Hamilton, M. D., upon the dietetics of nervous and mental diseases; by John V. Shoemaker, M. D., upon the relation of diet to personal beauty; by J. N. Love, M. D. (editorial), upon "diet in diphtheria, with special consideration of the proper food for children."

These articles show the general scope of the journal, and we cordially recommend it to our readers. W. M. J.

